# Exploiting geometric similarity for statistical quantification of fluorescence spatial patterns in bacterial colonies

**DOI:** 10.1186/s12859-020-3490-1

**Published:** 2020-06-03

**Authors:** David R. Espeso, Elena Algar, Esteban Martínez-García, Víctor de Lorenzo

**Affiliations:** Systems Biology Program, Centro Nacional de Biotecnología (CNB-CSIC), Campus de Cantoblanco, 28049 Madrid, Spain

**Keywords:** CSLM, Software, Bacteria, Geometric similarity, Colonies, Statistical analysis, Spatial distribution, Pattern, GFP

## Abstract

**Background:**

Currently the combination of molecular tools, imaging techniques and analysis software offer the possibility of studying gene activity through the use of fluorescent reporters and infer its distribution within complex biological three-dimensional structures. For example, the use of Confocal Scanning Laser Microscopy (CSLM) is a regularly-used approach to visually inspect the spatial distribution of a fluorescent signal. Although a plethora of generalist imaging software is available to analyze experimental pictures, the development of tailor-made software for every specific problem is still the most straightforward approach to perform the best possible image analysis. In this manuscript, we focused on developing a simple methodology to satisfy one particular need: automated processing and analysis of CSLM image stacks to generate 3D fluorescence profiles showing the average distribution detected in bacterial colonies grown in different experimental conditions for comparison purposes.

**Results:**

The presented method processes batches of CSLM stacks containing three-dimensional images of an arbitrary number of colonies. Quasi-circular colonies are identified, filtered and projected onto a normalized orthogonal coordinate system, where a numerical interpolation is performed to obtain fluorescence values within a spatially fixed grid. A statistically representative three-dimensional fluorescent pattern is then generated from this data, allowing for standardized fluorescence analysis regardless of variability in colony size. The proposed methodology was evaluated by analyzing fluorescence from GFP expression subject to regulation by a stress-inducible promoter.

**Conclusions:**

This method provides a statistically reliable spatial distribution profile of fluorescence detected in analyzed samples, helping the researcher to establish general correlations between gene expression and spatial allocation under differential experimental regimes. The described methodology was coded into a MATLAB script and shared under an open source license to make it accessible to the whole community.

## Background

The combination of new optical visualization techniques that use fluorophores to study gene expression with efficient algorithms to analyze data has been pushing synthetic biology to new levels [1]. Recent software and hardware developments are increasing the analysis capabilities of researchers, providing them with enhanced accuracy and specificity when studying gene expression within complex populations [2, 3], differentiation of subpopulations in microbial colonies [4, 5] or spatially location and inspection of areas of interest at individual cell level [6], among other uses. Despite the growing tendency in biology to rely upon imaging analysis software, there are still various fields in which use of such software is not wide spread [1]. Often, this resistance is due to researchers not finding a software package that effectively responds to their needs: many software tools were initially developed to deal with specific problems in a certain field and thus are tightly fitted to that field of study [7]. These software programs are then further expanded in a generalist fashion to adapt to a broader user community, not taking into account the specific needs of every potential user [8, 9]. This situation suggests that, although very powerful software does currently exist, tailored software is still an essential component for meeting more specific needs of many researchers.

An example of the need for more tailored software is found in the study of microbial colonies by microbiologists and biophysicists, where the spatial allocation of fluorescent regions (associated with extra- or intracellular probes) is essential for analyses of processes such as morphogenesis [10, 11], cellular differentiation [4] or the physical-chemical conditions affecting the development of multicellular communities [12, 13]. These studies are strongly limited by the intrinsic morphological variability associated with the cellular growth process, which requires manual analysis of data. Case studies where physical arrangement of bodies exhibits randomness or fractal patterning (i.e. neuron development [14, 15] and fungal fruiting bodies [16]) involves an additional level of difficulty due to the vast structural heterogeneity displayed (thus hindering the systematic collection of measurements for statistical purposes, as well as the ability to generalize results). Nevertheless, in cases where morphogenetic processes lead to a set of geometrically similar structures which can be systematically transformed onto a common reference frame, structural tendencies of pattern formation can be studied at population level.

In this work a specific methodology is presented to systematize the gathering and analysis of bacterial colonies exhibiting circular symmetry, despite variations in size and depth of samples.

## Results

### Rationale

The proposed methodology is based on exploiting the geometric similarity that bacterial colonies (3–6 days of growth) normally exhibit, uniformizing their shapes by applying similarity transformations, a subset of a broader group of operations termed affine transformations [17]. Recall that a similarity transformation is any mapping function that preserves not only collinearity, parallelism, convexity, and ratios of distances among parallel lines (common characteristics to all affine transformations), but also angles and proportionality (specific of that subgroup). As a result, transformed objects are *similar* to the original body (they resemble the same shape, angles and proportion, according to a ratio of magnification [17]). Specifically, uniform scaling, rotation and translation are the applied operations in this methodology.

**Fig. 1:**
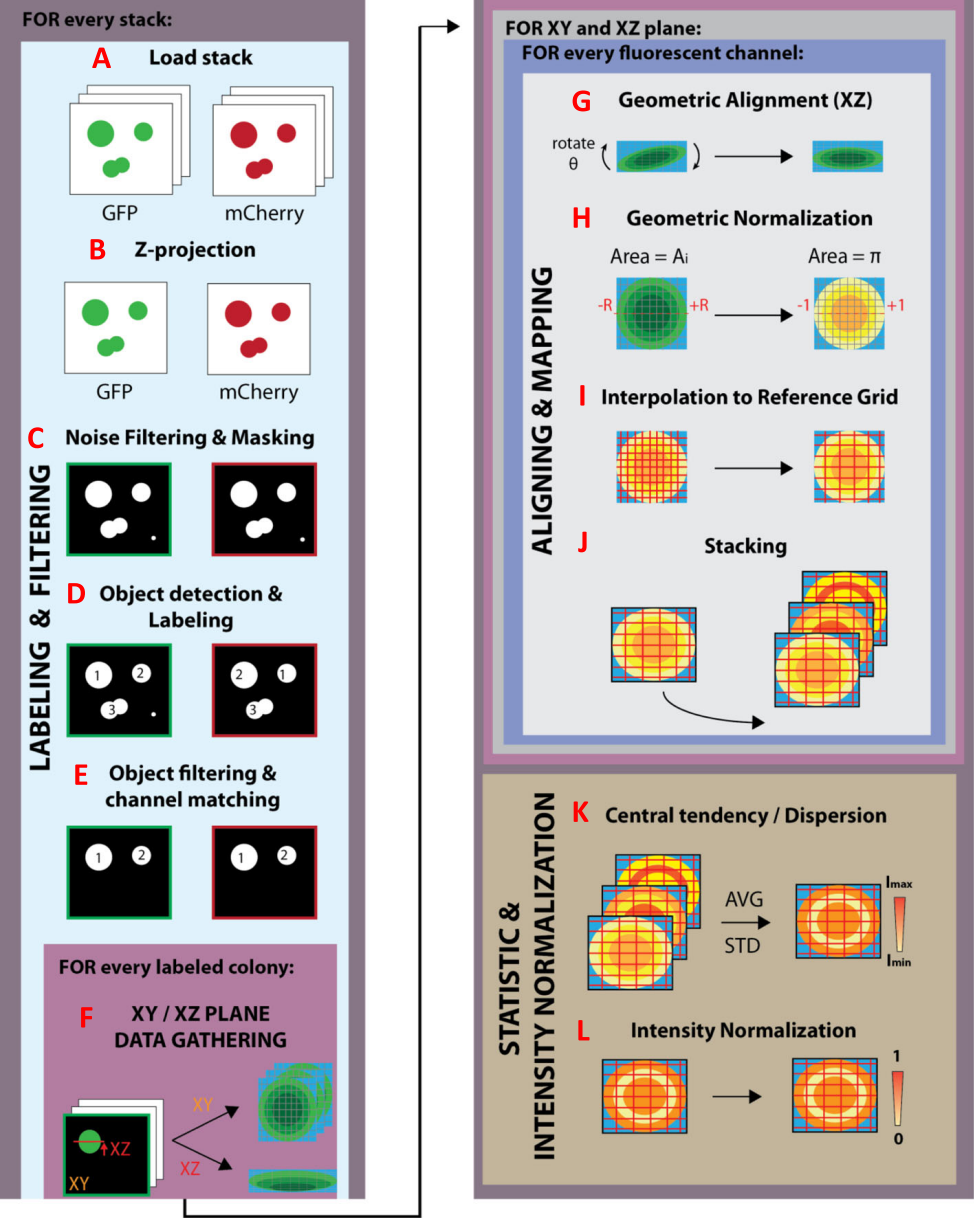
Schematic workflow of the proposed methodology. Raw images are first loaded, spatially delimited, labeled and filtered (**a**-**e**). Next for every labeled colony, the profiles are aligned and geometrically normalized (**f**-**h**) prior to interpolation of the intensity values in a reference grid (**i**). Profiles are recurrently stored to perform statistics in either raw or normalized units (**j**-**l**)

The circular symmetry and narrowly bounded variability in axial direction allow for the establishment of a computational workflow that applies a systematic set of filters and transformations, which are depicted in Fig. 1. This allows a mapping from a physical coordinate system to a normalized dimensionless reference frame where spatial positioning among replicas is comparable for statistical purposes. The specifics of this workflow are described next.

### Labeling and filtering

Images are first loaded into memory (Fig. 1a). The analysis pipeline then starts with a selection and filtering stage of all colonies present in the images. To clearly delimit the boundaries of individual colonies, two independent fluorescent reporters (GFP and mCherry) were used to, respectively, monitor the activity of the promoter and spatially locate colony boundary. The filtering process was based on discriminating circular objects from top-view images containing an arbitrary number of colonies. The first step to automate colony detection consisted on applying the XY sum projection (**Proy**) in both fluorescent channels along axial direction (z-stack) to obtain two single planar images (one for each channel), that further served as a stencil for object detection (Fig. 1b). Thus:

$$ \mathbf{Proy}=\sum \limits_{\mathrm{z}=1}^{{\mathrm{Z}}_{\mathrm{stack}}}{\mathbf{S}}^{\mathrm{z}} $$

where **S**^z^ is a three-dimensional matrix of size P_x_ x P_y_ x Z_stack_ containing the whole Z-stack image, Z_stack_ is the number of Z-planes in the stack, P_x_ and P_y_ are the pixel resolution of the gathered image and **Proy** is the sum projection matrix (size P_x_ x P_y_) in the Z direction.

A binarization stage was applied to discard the background of the image (using as a threshold value the average intensity of the image multiplied by a factor of 1.1), generating a boolean mask wherein pixel values were either 1 or 0 (Fig. 1c). We define this threshold as *Th* in order to create a filtering operator F(**X**) that drops values of X smaller than *Th*:

1 $$ Th=1.10\cdot \frac{1}{P_x\cdot {P}_y}\sum \limits_{i=1}^{P_x}\sum \limits_{j=1}^{P_y}{\mathrm{Proy}}_{\boldsymbol{ij}} $$

2 $$ F\left(\mathbf{X}\right)=\Big\{{\displaystyle \begin{array}{c}\kern0.50em {\mathrm{X}}_{ij}\kern0.1cm \mathrm{i}\mathrm{f}\kern0.1cm {\mathrm{X}}_{ij}>\mathrm{Th}\\ {}0\kern0.1cm \mathrm{otherwise}\end{array}}\kern0.3cm \mathrm{i}\mathbf{\in}\left[1,{\mathrm{P}}_x\right],\kern0.1cm \mathrm{j}\mathbf{\in}\left[1,{\mathrm{P}}_{\mathrm{y}}\right],\kern0.2cm \mathrm{i},\mathrm{j}\in \mathbb{N}\operatorname{} $$

where Proy_ij_ are the elements of the **Proy** matrix, Th is the threshold value to filter and F(**X**) is the filtering criteria applied to every element X_ij_ of matrix $\mathbf{X}\boldsymbol{\in }{\mathbf{\mathcal{M}}}_{{\boldsymbol{P}}_{\boldsymbol{x}}\times {\boldsymbol{P}}_{\boldsymbol{y}}}\left(\mathbb{R}\right)$. The binary operator Bin(**F**) is next applied to the filtered projection matrix **ProyF** to create Boolean mask **B**:

$$ \mathbf{ProyF}=F\left(\mathbf{Proy}\right) $$

3 $$ Bin\left(\mathbf{X}\right)=\Big\{{\displaystyle \begin{array}{c}1\kern0.1cm \mathrm{i}\mathrm{f}\kern0.1cm {\mathrm{X}}_{ij}>0\\ {}0\kern0.1cm \mathrm{otherwise}\end{array}}\kern0.3cm \mathrm{i}\mathbf{\in}\left[1,{\mathrm{P}}_x\right],\kern0.1cm \mathrm{j}\mathbf{\in}\left[1,{\mathrm{P}}_{\mathrm{y}}\right],\kern0.2cm \mathrm{i},\mathrm{j}\in \mathbb{N}\operatorname{} $$

$$ \mathbf{B}=\mathrm{Bin}\left(\mathbf{ProyF}\right) $$

Binary connected components within **B** matrices were detected and labeled using the algorithm described by Haralik and Shapiro [18] (Fig. 1d). Each object was treated as an array of pixels ${\mathbf{b}}^{\boldsymbol{n}}\boldsymbol{\in}{\mathbf{\mathcal{M}}}_{\mathbf{1}\times {\mathrm{N}}_{\mathrm{pixel}}^{\mathrm{n}}}\left(\mathbb{N}\right)$ with value 1 whose respective row and column indices are given in two vectors: ${\mathbf{bx}}^{\boldsymbol{n}}\boldsymbol{\in}{\mathbf{\mathcal{M}}}_{\mathbf{1}\times {\mathrm{N}}_{\mathrm{pixel}}^{\mathrm{n}}}\left(\mathbb{N}\right)$ and ${\mathbf{by}}^{\boldsymbol{n}}\boldsymbol{\in}{\mathbf{\mathcal{M}}}_{\mathbf{1}\times {\mathrm{N}}_{\mathrm{pixel}}^{\mathrm{n}}}\left(\mathbb{N}\right)$ respectively. XY object area (A) and mass center for every detected object were calculated by computing their zeroth and first moments as follows:

$$ {A}_n={\mathrm{N}}_{\mathrm{pixel}}^{\mathrm{n}} $$

$$ {\boldsymbol{CM}}_n=\left(\frac{1}{{\mathrm{N}}_{\mathrm{pixel}}^{\mathrm{n}}}\sum \limits_{i=1}^{N_{pixel}}{bx}_i^n,,,\frac{1}{{\mathrm{N}}_{\mathrm{pixel}}^{\mathrm{n}}}\sum \limits_{i=1}^{N_{pixel}}{by}_i^n\right) $$

where *n* represents each individual object detected in the image, ${\mathrm{N}}_{\mathrm{pixel}}^{\mathrm{n}}$ is the number of pixels detected in object *n* and CM_n_ is the point position in the XY plane (in pixel units) of the mass center of object n.

Detected elements with a XY area smaller than an empirically chosen value (an equivalent circular area of 20 pixels in the present case) were discarded. Major and minor axis lengths in the XY plane for each object *n* were derived from maximum and minimum detected X and Y values found within **b**_x_^n^ and **b**_y_^n^. At this point those elements having a major-minor axis length ratio larger than 15% were also excluded to avoid non-circular geometries (e.g. merged colonies, Fig. 1e).

Performing a point-by-point transposition of the fluorescence distribution into a three-dimensional body is quite complex because it is impossible to observe all points simultaneously. Instead it is more advisable to use fixed planes to study the distribution of the signal inside the whole body. Although this is tricky for bodies with arbitrary geometry, for cases with circular symmetry a very convenient choice is to inspect an axial projection (XY) and radial cross-section (XZ): the former shows the trend of the studied signal for increasing radial distances, while the later depicts the behavior along the Z axis in a diametric plane. Thus, XY and XZ planes were gathered from every colony (Fig. 1f).

### Aligning and mapping

Prior to mapping the data, every image was geometrically aligned. XY projections did not need any adjustment because all colonies exhibit circular symmetry. However, XZ images needed to be horizontally aligned due to the presence of imperfections on the agar surface, variability among samples and the natural curvature of agar when it is close to the edge of the culture plate. XZ profiles were realigned along the X axis by obtaining the orientation of the major axis with respect to the X axis (given by the angle θ between both axis, see [19] for more details) and then rotating the point coordinates of all pixels using a rotation matrix (Fig. 1g). So where **P**^m^_xz_ and **P**^m^_xz_**’** are, respectively, the unaligned and aligned point coordinates of the chosen radial cross-section *m* (with length 2 x N^m^_pixel_), they relate by means of the following rotation matrix around the Y axis:

$$ {{\boldsymbol{P}}_{\boldsymbol{XZ}}^m}^{\prime }=R\left(\theta \right)\cdot {\boldsymbol{P}}_{\boldsymbol{XZ}}^m=\left(\begin{array}{cc}\cos \left(\theta \right)& -\sin \left(\theta \right)\\ {}\sin \left(\theta \right)& \cos \left(\theta \right)\end{array}\right)\cdot {\boldsymbol{P}}_{\boldsymbol{XZ}}^m $$

As mentioned previously, to respond to colony size variability, a mapping process was applied to the XY and XZ planes of every colony. Data was transformed from a physical dimension-based reference frame to a normalized XYZ domain bounded within X ∈ [−1, 1], Y ∈ [−1, 1], Z ∈ [0, 1], using as normalizing dimensions the radius (for XY projection) and maximum height (for XZ plane) of each colony (Fig. 1h). This is equivalent to rescaling the spatial dimensions of all colonies to a similar size. We first compute the radius *R*, the height *H* and the minimum height *h* (defined as being where the base of the colony lays) as follows:

$$ R=\underset{n_1}{\max}\left\{\underset{x,y}{\max },\left\{{{\boldsymbol{P}}_{\boldsymbol{XY}}^m}_{\kern0.1cm n}^{\prime }-{\boldsymbol{CM}}_{XY}^m\right\}\right\} $$

$$ H=\underset{n_2}{\max}\left\{\underset{z}{\max },\left\{{{\boldsymbol{P}}_{\boldsymbol{XZ}}^m}_{\kern0.1cm n}^{\prime}\right\},-,\underset{z}{\min },\left\{{{\boldsymbol{P}}_{\boldsymbol{XZ}}^m}_{\kern0.1cm n}^{\prime}\right\}\right\} $$

4 $$ h=\underset{z}{\min}\left\{{{\boldsymbol{P}}_{\boldsymbol{XZ}}^m}_{\kern0.1cm n}^{\prime}\right\} $$

where we make an abuse of notation to indicate that maximum and minimum operator are applied only on x, y or z components of points **P**^m^_XY_^’^_n_ and **P**^m^_XZ_^’^_n_. *n*_*1*_ and *n*_*2*_ are the number of points forming the XY and XZ planes, respectively. **CM**^m^_XY_ and **CM**^m^_XZ_ are the mass centers of the XY and XZ planes (calculated as previously described). These three variables are then used to normalize all points:

$$ {{\hat{{P}}}_{XYn}^{m{\prime}}}=\left(\frac{{\left({{P}}_{XY}^m{\prime}_{n}\right)}_x-{\left({{CM}}_{XY}^m\right)}_x}{\mathrm{R}},\frac{{\left({{P}}_{XY}^m{\prime}_{n}\right)}_y-{\left({{CM}}_{XY}^m\right)}_y}{\mathrm{R}}\right) $$

$$ {{\hat{\textbf{P}}}^{m{\prime}}}_{XZn}=\left(\frac{{\left({\textbf{P}}_{XZ}^m{\prime}_{n}\right)}_x-{\left({\textbf{CM}}_{XZ}^m\right)}_x}{\mathrm{R}},\frac{{\left({\textbf{P}}_{XZ}^m{\prime}_{\kern0.05cm n}\right)}_{\mathrm{z}}-h}{\mathrm{H}}\right) $$

here ()_x_ and ()_z_ denote the x and z components of the considered points, and the hat operator is used to design normalized versions of rotated points.

To spatially correlate position and signal, cell locations need to be fixed in a reference grid. An experimental solution to this issue would be extremely complex due to natural replica variability. Nevertheless, it is still possible to numerically overcome this problem by using data interpolation to estimate the values of signal intensity for every colony within a fixed grid of coordinates, using experimental data from arbitrary positions throughout the colony (Fig. 1i). The interpolant grid should have a density point smaller than the original image resolution to minimize lack of data when estimating values. In this work two grids of smaller resolution ([256 × 256] points for XY projections and [256 × 10] points for XZ planes) were used to cover the normalized domain. A barycentric-based coordinate cubic interpolation algorithm supported by a Delaunay triangulation of the pixel coordinates was chosen to estimate values [20]. Interpolated profiles were finally stored in a sequential manner to create a stack of profiles (Fig. 1j) from which statistical measurements were performed.

### Intensity normalization

Stored XY and XZ profiles were used to estimate the central tendency of the intensity distribution for every set of experimental conditions and in every fluorescent channel (mean, median, see Fig. 1k). Depending on a researcher’s needs, intensity values either can be handled as raw data or can be first normalized with respect to maximum and minimum reference values (Fig. 1l). Raw values can be used to establish fold-change comparison of measurements among samples using a semibounded scale ([0, ∞)), provided that all samples are gathered with the same microscope settings. Normalized data can be used to locate heat areas of intensity signal in the colony and local variations within colonies when microscope settings cannot be standardized among samples. The most common option to normalize data is to work with positive / negative control samples to perform the transformation:

$$ {\mathbf{I}}^{\ast }=\frac{{\mathbf{I}}_M-{\mathbf{I}}_{{\mathrm{C}}^{-}}}{{\mathbf{I}}_{{\mathrm{C}}^{+}}-{\mathbf{I}}_{{\mathrm{C}}^{-}}} $$

where I* and **I** are, respectively, normalized and non-normalized intensity matrices, and M, C^+^ and C^−^ subscripts denote the sources of the samples (regular sample, positive control and negative control respectively).

Unfortunately, there are circumstances in which any one of these controls may not be suitable to use due to modification microscopy settings to avoid image acquisition quality problems (i.e. signal saturation due to the existence of large differences in intensity values between samples and the positive control). An alternative choice for these cases is to scale values by using the initial maximum and minimum intensity values found in every profile, as follows:

$$ {\mathbf{I}}_{\mathrm{XY}}^{\ast }=\frac{{\mathbf{I}}_{\mathrm{XY}}-\min \left({\mathbf{I}}_{\mathrm{XY}}\right)}{\max \left({\mathbf{I}}_{\mathrm{XY}}\right)-\min \left({\mathbf{I}}_{\mathrm{XY}}\right)} $$

$$ {\mathbf{I}}_{\mathrm{XZ}}^{\ast }=\frac{{\mathbf{I}}_{\mathrm{XZ}}-\min \left({\mathbf{I}}_{\mathrm{XZ}}\right)}{\max \left({\mathbf{I}}_{\mathrm{XZ}}\right)-\min \left({\mathbf{I}}_{\mathrm{XZ}}\right)} $$

where I^*^_XY_ and I^*^_XZ_ are the XY and XZ-normalized intensity profiles for each experimental condition and fluorescent channel, **I**_XY_ and **I**_XZ_ are the absolute intensity profiles, and max (**I**_XY_), max(**I**_XZ_), min(**I**_XY_), min(**I**_XZ_) are the maximum and minimum values of intensity detected in **I**_XY_ and **I**_XZ_ respectively for the chosen experimental conditions.

### Experimental validation

**Fig. 2:**
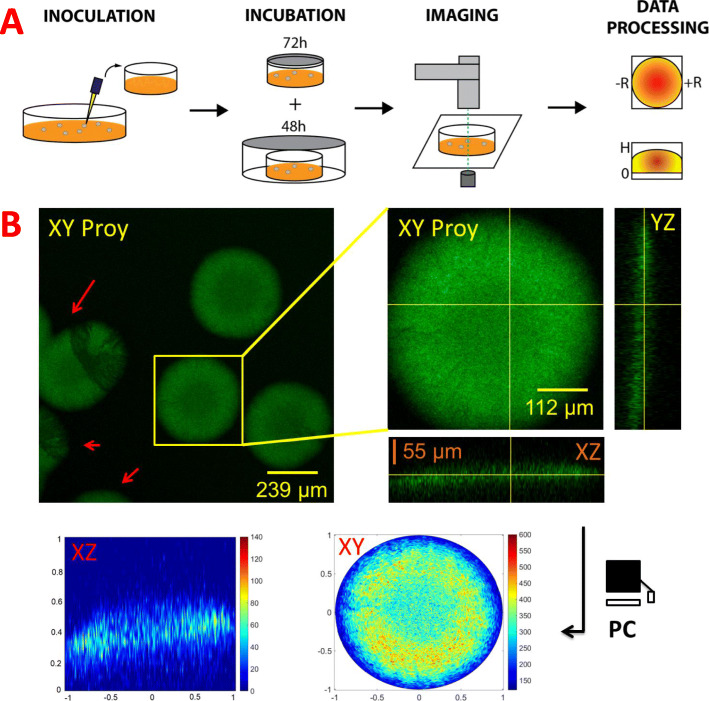
Experimental methodology (**a**) and numerical analysis of gathered images (**b**) applied to obtain the analyzed data. Monoclonal colonies were picked and streaked onto a 60 mm culture dish, followed by an incubation of 5 days prior to the acquisition and further analysis of every colony within the images. The analysis provided statistically comparable data for all the colonies

In order to validate the proposed methodology, we performed a growth experiment on agar plates using a *P. putida* KT2440-mCherry strain carrying a plasmid that produces GFP as regulated by a promoter whose expression varies with spatial position within the colony. In this case we chose a promoter that has been reported to respond to environmental humidity [in preparation], thus colonies exhibit a spatial fluorescence pattern according to water access within the colony. The experimental procedure followed is detailed in the methods section and depicted in Figs. 2a (experiment) and 2B (image analysis). To summarize this procedure in brief: individual bacterial colonies were streaked onto 60-mm culture dishes and incubated at 30 °C for 5 days, letting the agar dry. Colonies were imaged using CSLM technique to generate Z-stack images that were sequentially analyzed to gather the fluorescence profile of every individual colony. These profiles were transformed to produce statistically treatable data.

**Fig. 3:**
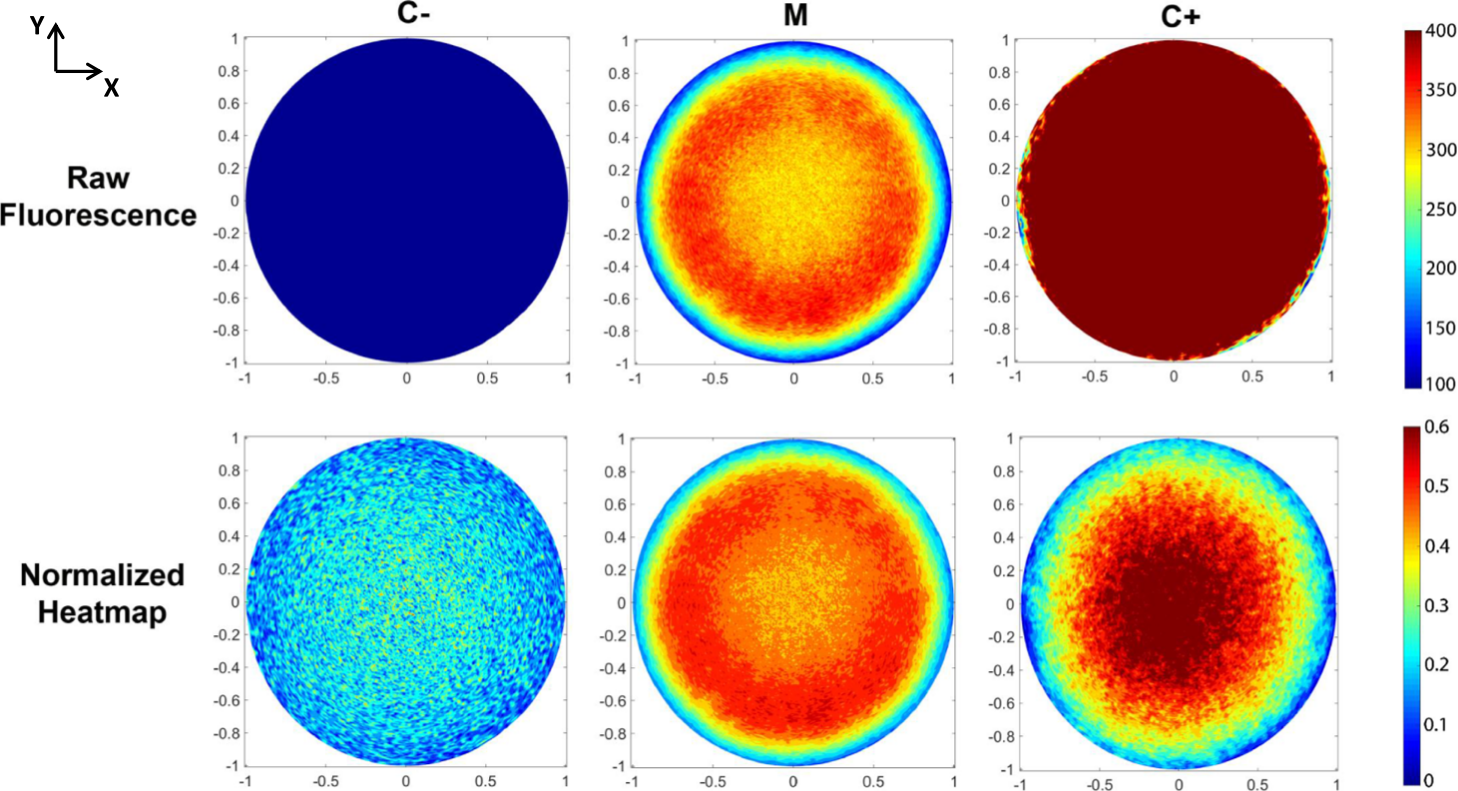
Raw fluorescence (up) and normalized heatmaps (down) obtained for the analyzed sample (M), as well as the positive (C+) and negative (C-) controls

**Fig. 4:**
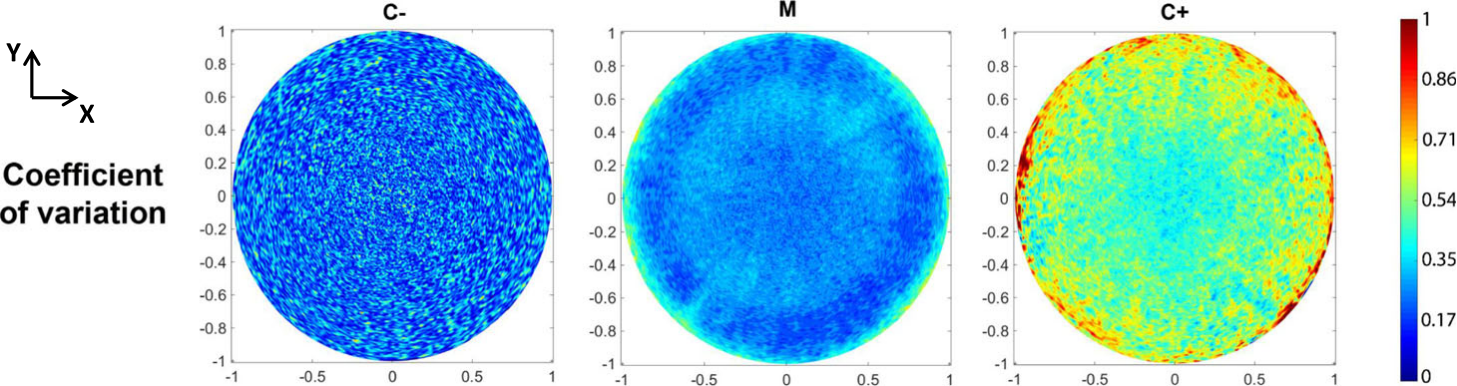
Variation coefficient (CV) obtained for different samples type (sample M, positive control C+ and negative control C-). CV values increase when the number of analyzed samples is small (see C+ sample) or when colonies are located at the boundaries where the interpolant algorithm tends to provide worse estimations

As a benchmark test, three sets of colonies were cultured: one carrying the desiccation-inducible promoter (the sample to analyze, named M), a chemically-inducible promoter (serving as a positive control, named C+) and a non-expressing plasmid (serving as a negative control, named C-). The results of the analysis are shown in Fig. 3 (XY projection) and Additional Files 1, 2 and 3 (XZ slices). Raw fluorescence analysis (Fig. 3 upper row) showed a spatially dependent behavior of sample M when compared with controls, as it exhibited a ring-shaped distribution. Values of C+ and C- are, respectively, above and below the range of intensities exhibited by sample M. The differences in magnitude of the observed intensity made a direct comparison of the signal spatial distributions impossible, so intensity normalization was applied to correct this effect. The resulting heatmaps (Fig. 3 lower row) exhibited differences in fluorescent pattern that suggest a proper functioning of the desiccation-responsive promoter. Sample C- exhibited a noisy distribution not associated to the measured biological reporter, but rather to unspecific phenomena (i.e. self-fluorescence). Fluorescence in sample C+ displayed a classical 2D Gaussian distribution, resembling the overall biomass distribution of the colony and implying an accumulation of constitutively expressed fluorescent protein in the center of the colony. Since colonies are expected to gradually dry out during the course of the experiment, sample M should show a spatially dependent fluorescence profile mirroring the water distribution within the colony. The presence of a ring-shaped pattern confirms that the reporter distribution is not spatially correlated with biomass distribution nor associated to an unspecific phenomenon, but rather follows a well-defined arrangement. The coefficient of variations derived from the statistical analysis (see Fig. 4) showed a moderate error bar size in all samples except in positive control C+, where errors are large due to a low number of analyzed colonies.

Although the generated evidence does not confirm a direct relationship between the humidity distribution and the observed ring-shaped fluorescence pattern, the non-arbitrary order of the fluorescence profile does confirm an interaction between the tested reporter and a spatially-dependent variable. This conclusion is enough to validate the proposed methodology as a reliable method to measure spatial distribution of fluorescent signal in colonies.

## Discussion

The presented methodology was developed to automate analysis of CSLM image stacks of circular bacterial colonies, the most frequently observed pattern when cells grow on solid media (1.5% agar, w/v) in regular laboratory conditions. Nevertheless, its potential use can be extended to any microorganism that develop colonies of circular morphology. The method is based not on the type of microorganism, but rather on taking advantage of the geometric similarity of analyzed colonies. This allows for routine application of a set of mathematical transformations to gather normalized data regardless of colony size. The application of this method to non-symmetric samples could also be possible, but with numeric adaptations to deal with its asymmetric shape provided that those samples are all geometrically similar. The required algorithmic adjustment to process highly irregular bodies would involve the processing of the data using more general analysis techniques: modal matching [21–23], moment based methods [24, 25], geometric hashing [26, 27] or pose clustering [28] could help to deal with complex geometries. Random geometries on the contrary (i.e. diffusion driven spreading [29, 30]) are not treatable using this approach, because mapping function cannot be computed to project data into the normalized grid.

There is additionally a major limitation in this approach when applying the numerical procedure to generate the mapped intensity profile. Ideally, all images should be taken at the largest possible resolution to provide the smallest possible pixel area: this allows the generation of interpolated intensity profiles with larger resolution and more accuracy, even in smaller interpolating grids [31]. An insufficient biological sampling or the use of low-resolution images may lead to poor accuracy during interpolation when computing values. Furthermore, as the method is based on performing interpolation to estimate values in fixed spatial positions, this methodology will provide worse estimations close to the boundaries, where colonies exhibit a larger degree of variability (see Fig. 4). If the number of processed samples is low (*n* < 10 in our methodology using a conservative criterion, as is the case in Fig. 4 sample *C+*), the standard deviation values may drastically increase. This effect can be partially diminished by improving the statistical sampling (i.e. increasing the number of samples to process) or by enhancing the quality of data (i.e. increasing the bit depth of the gathered image), as the most straightforward alternatives to overcome the issue among others related with sampling process [32]. A computational approach based in replacing the interpolant algorithm (i.e. use of Radial Basis Functions [33]) can increase the precision of predictions in the grid of evaluation if required. Thus special attention must be paid when designing the experiments and computing each case to minimize these issues.

## Conclusion

The statistical study of fluorescent reporter distribution within bacterial colonies is cumbersome because of colony size variability (in both area and thickness) among samples, and the need to acquire data from a sufficient number of replicas to ensure reliable statistics. In this work a specific methodology was implemented in a MATLAB script in order to automate the selection and extraction of useful data from CSLM stack images of circular bacterial colonies. The process is computationally performed to allow an image analysis of all colonies independent of size variability. The methodology was experimentally validated by comparing the distribution of fluorescence exhibited by *P. putida* colonies carrying a plasmid regulated by a humidity-sensitive promoter. As the proposed numerical procedure exploits the geometric similarity of measured bodies and uses an interpolating approach to generate statistically comparable data, there are limitations when working with a low number of samples, poor quality images or non-symmetric bodies. Despite these limitations, the proposed approach offers a powerful and simple framework to study signal distribution of CSLM images in an automated and statistically reliable fashion.

## Methods

### Strains, plasmids media and growth conditions

The bacterial strain used here is a derivative of *P. putida* KT2440 with an mCherry fluorescent cassette integrated in its genome that constitutively expresses the red fluorescent protein. This fluorescent signal was used to locate the colony boundaries to perform the image processing [34]. This strain was transformed with three versions of the plasmid pGLR2, which contains a promoterless dual GFP-*luxCDABE* reporter system [35]. The three plasmids used here are: i) pGLR2 (a plasmid without promoter that does not fluoresce) used as a negative control; ii) pGLR2-P*trc* (with an IPTG-inducible promoter controlling fluorescence expression) used as a positive control; and iii) pGLR2-P_*4707*_ (with a humidity-sensitive promoter controlling the fluorescence [in preparation]) as the experimental sample. All the strains were grown overnight on regular M9 minimal medium Petri dishes [36] solidified with 1.5% (w/v) agar and amended with 0.2% (w/v) glucose, 1 mM IPTG. Samples were supplemented when required with 50 μg/ml Kanamycin and 15 μg/ml Gentamycin. Individual colonies were re-streaked to microscope-compatible 35 × 14 mm culture dishes (Ibidi) containing 2 ml of M9-agar with glucose as the carbon source (prepared as mentioned before) and incubated at 30 °C during 5 days (120 h). The first 72 h, all dishes were covered with individual lids. For the remaining 48 h, the dishes were incubated without their own lids but within a 92 × 16 mm Petri dish to promote a higher drying of the growing media. Relative humidity was not controlled during the experiment.

### Imaging acquisition

Colony images were gathered using a Confocal Multispectral Leica SP5 system with a HCX PL APO CS 10 × 0.40 DRY UV objective using 488 and 561 nm laser lines to detect GFP and mCherry fluorescent signals, respectively. Images were captured at 8-bit resolution (1024 × 1024) with no amplification factor and a frequency rate of 400 Hz. Distance between XY pixels and gathered Z planes included in stack images were 1.5137 and 6 μm, respectively. The numerical method was implemented in a script written in MATLAB (The Mathworks) containing the imaging toolbox and the MATLAB compatible bioformats package (https://docs.openmicroscopy.org/bio-formats/5.9.2/users/matlab/index.html#) on a regular PC. Confocal images shown in the manuscript were treated to enhance brightness and contrast using ImageJ software.

### Statistics

Experimental data gathered from different conditions was retrieved from two biological replicas, with at least 3 images for every condition. The number of processed colonies varied depending on the filtering criteria and the quality of the gathered image. For the parameters used in this manuscript, the final number of analyzed colonies was 21 for the humidity-sensitive strain, 5 colonies for the positive control and 3 colonies for the negative control.

## Supplementary information

**Additional file 1.** Raw fluorescence profiles for a XZ section (Y=0 plane) of monitored promoter (M), 4 positive control (C+) and negative control (C-).

**Additional file 2.** Normalized fluorescence profiles for a XZ section (Y=0 plane) of monitored promoter (M), positive control (C+) and negative control (C-)

**Additional file 3.** Variation coefficient for a XZ section (Y=0 plane) of monitored promoter (M), positive control (C+) and negative control (C-).

## Data Availability

A copy of the software together with CSLM images analyzed within this manuscript can be found in: https://fairdomhub.org/data_files/2946?version=1. The software can be found also in http://github.com/drespeso/GEOSIMCO. Operative system: Tested under Windows. Programming Language: Matlab. Other requirements: Image Processing Toolbox for Matlab, MATLAB compatible bioformats package. License: MIT.

## Abbreviations

CSLM: Confocal Scanning Laser Microscopy
IPTG: Isopropyl β-D-1-thiogalactopyranoside
