# Supplementary material for: Exploiting geometric similarity for statistical quantification of fluorescence spatial patterns in bacterial colonies
Source: BMC Bioinformatics. 2020 Jun 3;21:224. doi: 10.1186/s12859-020-3490-1 (PMC7268344; doi:10.1186/s12859-020-3490-1)
Supplement: Supplementary file 1 — Additional file 1. Raw fluorescence profiles for a XZ section (Y=0 plane) of monitored promoter (M), 4 positive control (C+) and negative control (C-). [file 12859_2020_3490_MOESM1_ESM.pdf]

Additional File 1

The results of the XZ plane (Y=0) analysis are presented below. Raw fluorescence, normalized heatmaps and coefficient of variations are provided. Results show a similar numerical trend that the one observed in XY plane. Raw fluorescence mean profiles exhibit a Switch ON / OFF color pattern in agreement with positive / negative control (C+ / C-) for any functional promoter. Sample M exhibit the ring pattern already mentioned in the main manuscript.

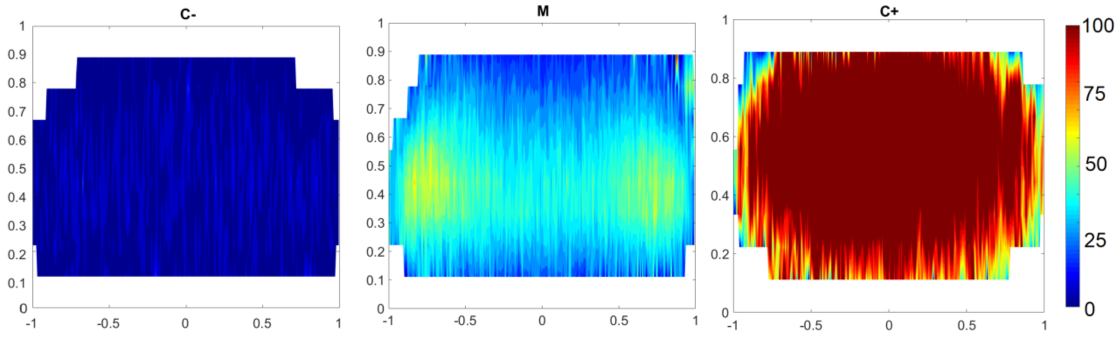

Raw fluorescence profiles for a XZ section (Y=0 plane) of monitored promoter (M), positive control (C+) and negative control (C-). Intensity scale has been adjusted to optimize the visualization of the ring pattern in M sample. Positive and negative controls exhibit intensity values above and below these values, showing the typical ON / OFF saturation profiles.

The normalized heatmaps also agree with reported results. Negative control do not exhibit any concrete pattern, positive control resembles the typical Gaussian profile with the hottest part in the center of the colony. M sample keeps showing the annular shape as in the case of the Raw fluorescence analysis.
