# Supplementary material for: Exploiting geometric similarity for statistical quantification of fluorescence spatial patterns in bacterial colonies
Source: BMC Bioinformatics. 2020 Jun 3;21:224. doi: 10.1186/s12859-020-3490-1 (PMC7268344; doi:10.1186/s12859-020-3490-1)
Supplement: Supplementary file 2 — Additional file 2. Normalized fluorescence profiles for a XZ section (Y=0 plane) of monitored promoter (M), positive control (C+) and negative control (C-) [file 12859_2020_3490_MOESM2_ESM.pdf]

Additional File 2

Normalized fluorescence profiles for a XZ section (Y=0 plane) of monitored promoter (M), positive control (C+) and negative control (C-).

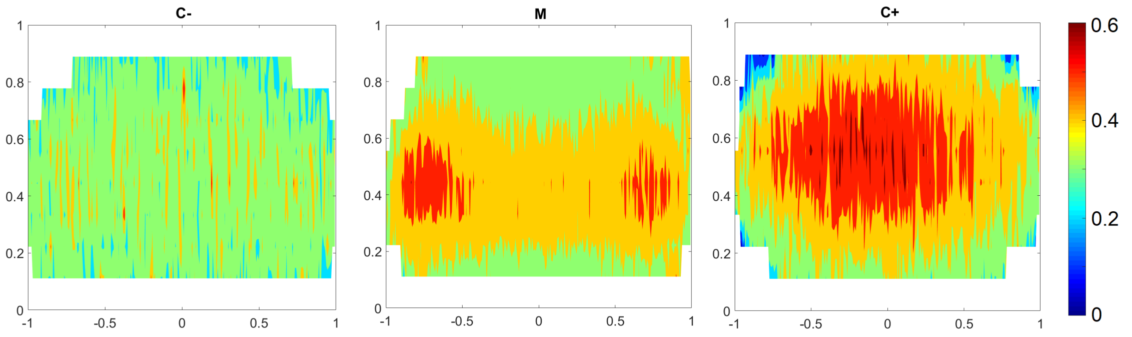

The monitored sample (M) exhibit a ring pattern as previously described. Negative control (C-) did not show any ordered distribution, and positive control (C+) showed a typically Gaussian distribution centered in the middle of the colony
