# Supplementary material for: Exploiting geometric similarity for statistical quantification of fluorescence spatial patterns in bacterial colonies
Source: BMC Bioinformatics. 2020 Jun 3;21:224. doi: 10.1186/s12859-020-3490-1 (PMC7268344; doi:10.1186/s12859-020-3490-1)
Supplement: Supplementary file 3 — Additional file 3. Variation coefficient for a XZ section (Y=0 plane) of monitored promoter (M), positive control (C+) and negative control (C-). [file 12859_2020_3490_MOESM3_ESM.pdf]

Additional File 3

Variation coefficient for a XZ section (Y=0 plane) of monitored promoter (M), positive control (C+) and negative control (C-).

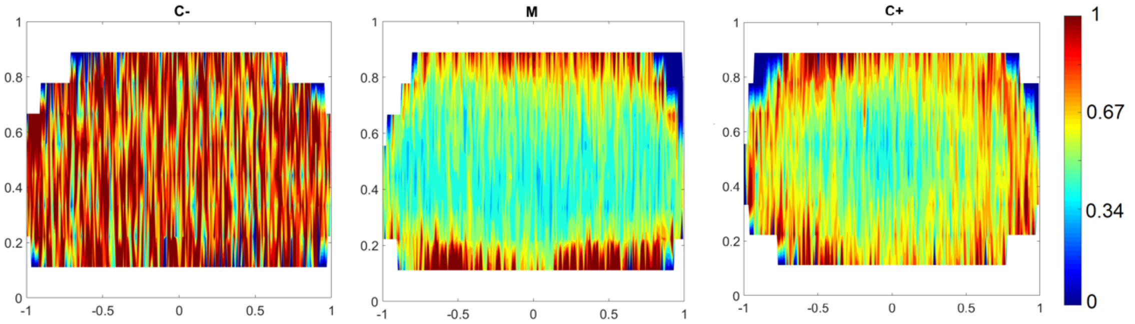

CV values are larger in those grid points close to the boundaries, where numerical interpolation is less accurate. Standard deviation is larger as the number of available repetitions for a sample decreases.
